# Changing Folding and Binding Stability in a Viral Coat Protein: A Comparison between Substitutions Accessible through Mutation and Those Fixed by Natural Selection

**DOI:** 10.1371/journal.pone.0112988

**Published:** 2014-11-18

**Authors:** Craig R. Miller, Kuo Hao Lee, Holly A. Wichman, F. Marty Ytreberg

**Affiliations:** 1 Department of Biological Sciences, University of Idaho, Moscow, Idaho; 2 Department of Mathematics, University of Idaho, Moscow, Idaho; 3 Institute for Bioinformatics and Evolutionary Studies, University of Idaho, Moscow, Idaho; 4 Department of Biochemistry and Molecular Biophysics, Kansas State University, Manhattan, Kansas; 5 Department of Physics, University of Idaho, Moscow, Idaho; Russian Academy of Sciences, Institute for Biological Instrumentation, Russian Federation

## Abstract

Previous studies have shown that most random amino acid substitutions destabilize protein folding (i.e. increase the folding free energy). No analogous studies have been carried out for protein-protein binding. Here we use a structure-based model of the major coat protein in a simple virus, bacteriophage φX174, to estimate the free energy of folding of a single coat protein and binding of five coat proteins within a pentameric unit. We confirm and extend previous work in finding that most accessible substitutions destabilize both protein folding and protein-protein binding. We compare the pool of accessible substitutions with those observed among the φX174-like wild phage and in experimental evolution with φX174. We find that observed substitutions have smaller effects on stability than expected by chance. An analysis of adaptations at high temperatures suggests that selection favors either substitutions with no effect on stability or those that simultaneously stabilize protein folding and slightly destabilize protein binding. We speculate that these mutations might involve adjusting the rate of capsid assembly. At normal laboratory temperature there is little evidence of directional selection. Finally, we show that cumulative changes in stability are highly variable; sometimes they are well beyond the bounds of single substitution changes and sometimes they are not. The variation leads us to conclude that phenotype selection acts on more than just stability. Instances of larger cumulative stability change (never via a single substitution despite their availability) lead us to conclude that selection views stability at a local, not a global, level.

## Introduction

Biological systems require proteins, and to function structured proteins require a minimum level of thermodynamic folding stability [1], [2]. Most functioning proteins are marginally stable, with a folding thermodynamic stability between −5 and −15 kcal/mol [3]–[7]. The thermodynamic folding stability is an equilibrium measure of the fraction of folded to unfolded proteins given by the Gibbs free energy difference of folding, *ΔG_fold_*, and can be experimentally determined by measuring the equilibrium constant [8]–[11]. Under equilibrium conditions, an increase in the thermodynamic folding stability of a protein corresponds to an increase in the fraction of time a protein is folded.

Protein folding stability can be broken down into several molecular interactions that depend on protein structure and environmental conditions [12]–[14]. Similarly, protein-protein binding stability, the equilibrium measure of the fraction of bound to unbound proteins, is also a function of these interactions. Hydrophobic interactions contribute to stability in proportion to the size of the protein and primarily tend to stabilize the globular conformation [3], [15], [16]. Increased temperature can reduce the hydrophobic effect and the tendency for protein association reactions become enthalpy dominated [1], [4], [17]–[20]. Burying polar residues contributes to folding stability since the intramolecular hydrogen bonding and van der Waals interactions of polar groups in folded proteins are more favorable than similar interactions with water in unfolded proteins [21], [22]. Changes in ion concentration or pH also alters the thermodynamic stability [23], [24].

There is often a tradeoff between protein stability and protein function because proteins that are too stable can be less functional [2], [19], [25], [26]. For example, a study of β-lactamase TEM-1 by Wang and collaborators showed that mutant enzymes with increased activity against antibiotics were less stable [27], [28]. Similarly, five key active-site residues of AmpC β-lactamase have been characterized as decreasing the activity and increasing the stability of the enzyme [20], [23], [29]. These studies illustrate how changes in protein stability can result in changes of functional enzymatic activity.

Random substitutions of globular proteins tend to destabilize folding by decreasing the thermodynamic folding stability. Bloom and collaborators presented a thermodynamic framework to predict the probability that a protein retains its structure after one or more random amino acid substitutions, and highly simplified models of proteins were used to support their prediction that the substitutions tend to be destabilizing [4], [7], [8], [15], [22], [23]. A study by Tawfik and collaborators showed that about 70% of random substitutions of globular proteins are destabilizing (*ΔΔG*>0 kcal/mol), and that about 20% are highly destabilizing (*ΔΔG*>2 kcal/mol) [15], [17], [24], [25]. In another study they found that substitutions associated with new enzymatic functions are mostly destabilizing [1]–[3], [5]–[7], [17], [19], [26], [27]. One reason that these findings are important is because it is thought that many monogenic diseases are caused, in part, by decreased protein thermodynamic stability [4], [8], [23], [30]–[32]. A typical disease-causing mutation destabilizes protein folding by increasing the folding free energy by 2–3 kcal/mol [9]–[12], [32], [33].

Understanding the effect of random amino acid substitutions on protein-protein binding is critical to understanding protein evolution as well as potentially elucidating the biophysical mechanisms for some diseases. Since proteins frequently bind to other proteins to function, we hypothesize that either over-stabilizing or destabilizing protein-protein binding may cause loss of biological function (consistent with the ideas in [2], [13]–[15], [34]–[37]). For example, it has been shown that mis-assembly of homomers (self-interacting copies of a protein unit) is implicated in diseases [1], [4], . One such disease is Parkinson's where the mis-assembly of protein complex I in brain mitochondria reduces the function of the complex [3], [5]–[7], [21], [39]. The effect of amino acid substitutions on the aggregation rates of unfolded polypeptides can be correlated to physicochemical properties, such as hydrophobicity, protein structure and electric charge distribution [23], [40], [41].

Studying how substitutions alter protein stability is also integral to understanding and even predicting how viral and bacterial infectious diseases or agricultural insect pathogens evolve in real time. We expect that a limited tolerance to changes in both binding and folding stability in turn constrain and influence the adaptive pathways available to these organisms. For example, substitutions that would be adaptive (e.g. by conferring a new function like metabolizing an antibiotic) may not be if they destabilize the protein too much. In such cases, otherwise neutral substitutions that happen to stabilize a protein may, by chance, preadapt it to tolerate this type of destabilizing gain-of-function mutation [20], [22], [23], [42]. Thus adaptation may not just be in response to direct selective forces; it may also be influenced circuitously by conditions like temperature and acidity that may select for changes in stability.

In this study, we determined how amino acid substitutions, accessible through a single mutation within a codon, change protein folding stability and protein-protein binding stability in a bacteriophage virus system. FoldX was used to estimate the changes in folding stability (*ΔΔG_fold_*) and binding stability (*ΔΔG_bind_*) for the coat protein F in the bacteriophage virus φX174 [7], [8], [12], [15], [24], [25]. Folding and binding stabilities were calculated for all accessible substitutions for each amino acid residue in the major capsid protein (F). We examined the distribution of all accessible effects. We then compared the accessible substitutions with those observed in real evolving phage: first among the wild φX174-like phage, and second in the context of laboratory adaptations of φX174 [2], [8], [15], [19], [26], [27], [41], [43]–[50]. We find that there are unexpected differences between accessible and observed substitutions. Observed substitutions tend to have smaller effects on stability than expected by chance. Substitutions observed in high temperature adaptations tend to stabilize folding but slightly destabilize binding. Finally their cumulative stability effects in lab adaptations can be considerably greater than individual effects suggesting that selection is acting on local aspects of protein stability.

## Results and Discussion

**Figure 1:**
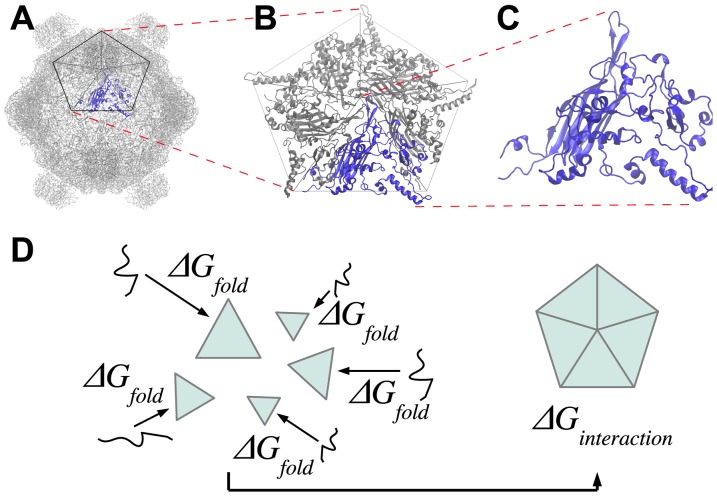
Model systems used in current study. (A) The capsid of φX174 consists of multiple copies of several kinds of proteins. The pentagon highlights a pentameric subunit that has five copies of coat protein F. (B) One pentameric subunit used in this study to estimate protein-protein binding stabilities, *ΔΔG_bind_*. (C) A single protein F used in this study to estimate protein folding stability, *ΔΔG_fold_*. (D) For each substitution within 1 DNA change of the reference sequence, we calculated *ΔΔG_fold_* and *ΔΔG_bind_* using FoldX and the conceptual model shown. For a given amino acid sequence of the F protein, we have *ΔG_bind_* = *ΔG_interaction_* – 5*ΔG_fold_*. Letting the subscripts *sub* and *ref* refer to the protein with and without a given substitution, the relative binding stability is then calculated as *ΔΔG_bind_* = *ΔG_bind,sub_ – ΔG_bind,ref_* and the relative folding stability is calculated as *ΔΔG_fold_* = *ΔΔG_fold,sub_ – ΔΔG_fold,ref_*.

The purpose of this study is examine the link between protein stability and natural selection by asking if and how substitutions fixed by selection differ from all accessible substitutions in their effects on both folding and binding stability. To do this we used the coat protein (protein F) from the phage φX174 as a model system (Figure 1A). As a first step in capsid formation in φX174, sets of five F proteins bind to form pentameric subunits (Figure 1B); twelve of these pentameric subunits then assemble in conjunction with several other proteins to form the capsid. We modeled the folding stability of individual F proteins (Figure 1C, 1D) and the binding stability of five folded mature F proteins into a single pentameric subunit (Figure 1B, 1D). More specifically, we used FoldX [1]–[3], [5]–[7], [15] to determine the effect on folding and binding stability of each amino acid change accessible within one DNA change from our reference sequence at every amino acid residue in the protein (Figure 1D). We choose this one DNA change criteria because nearly all the observed substitutions (discussed next) were within one DNA change. Stability effects were based on differences from our laboratory strain of φX174 (GenBank accession number AF176034 [4], [8]) at 37°C and expressed as *ΔΔG* in units of kcal/mol. Substitutions fixed by natural selection came from two sources: (1) differences observed among wild phage that are closely related to φX174 [9]–[12], and (2) substitutions observed among 26 laboratory adaptation experiments using φX174 [13]–[15].

The resolutions of the protein structure used for this study is 3.0 Å. It is known that the FoldX folding and binding stability results are more accurate for high resolution structures (<1.8 Å) [51]. There is, however, no evidence that FoldX shows systematic bias for low resolution structures. Statistical methods that have high variance have lower power, or a reduced probability of detecting effects that exists. But if they are unbiased, they do not suffer from an elevated risk of false discoveries (or type I errors). We believe the use of FoldX in the current study is analogous: using a low resolution structure may have reduced our predictive power but it should not have elevated our type I error rate. Thus the significant differences we uncover despite this reduced power would likely be even more strongly supported if structure resolutions were higher.

**Figure 2:**
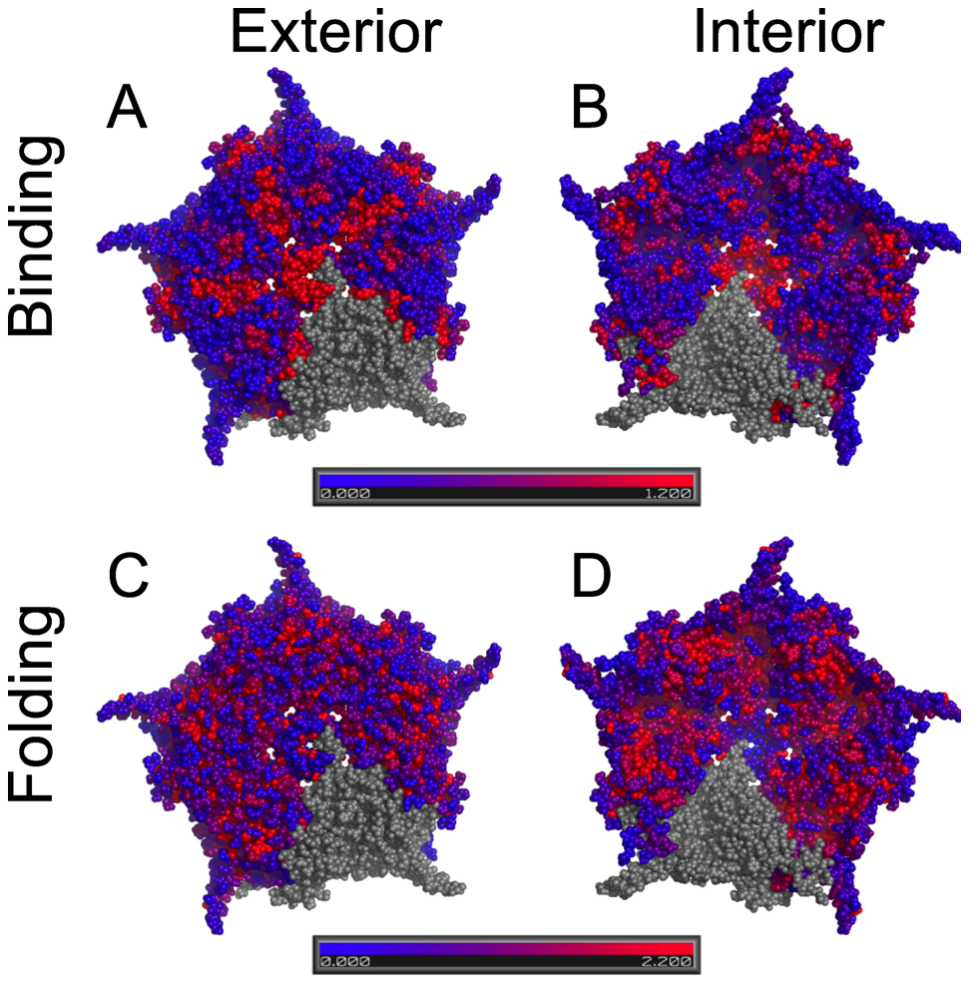
Heatmap of one pentamer showing median absolute effect size (i.e. |*ΔΔG*|) at each residue among accessible substitutions. The figure illustrates that for binding stability, high effect residues are found along protein-protein interfaces while for folding stability, high effect residues are concentrated in the interior of the protein. Residues in red have large median effects; those in blue have small effects. Top panels (A and B) show effects on binding stability while lower two panels (C and D) show effects on folding stability. Left panels (A and C) show the exterior surface; right panels (B and D) show interior surface.

As a method of evaluating whether our FoldX calculations are behaving as expected, we calculated the median effect on *ΔΔG_fold_* and *ΔΔG_bind_* of accessible substitutions at each residue. We then created heatmaps of the pentamer showing large median effects in red and low effects in blue. Since substitutions in residues along protein-protein interfaces have the potential to dramatically alter binding stability whereas residues far from an interface do not, we expect interface sites to show much larger binding effects. This is exactly what we observe (Figure 2A–B). By contrast, residues within the protein have more opportunity to interact with other residues of the same protein, leading us to expect that large-effect folding sites should be concentrated in the protein's interior and to thus have a very different pattern than binding effects. Again, this is what we observe (Figure 2C–D).

### Patterns Among Accessible Substitutions

When we examine the effect of all substitutions within one DNA change, our results indicate that most accessible substitutions destabilize both folding and binding. For folding, 72.9% of the accessible substitutions have *ΔΔG_fold_*>0. This agrees with previous studies that have shown random substitutions tend to be destabilizing [1], [4], [16], [17], . We also find that a majority of accessible substitutions destabilize binding since 70.0% of the accessible substitutions have *ΔΔG_bind_*>0. Note that 70% reflects destabilization of a single pentamer; in an expanded model that included multiple pentamers and interactions of the coat protein with other capsid proteins, we would expect this value would be higher. This prediction is supported by the graphic representation shown in Figure 2 where substitutions with moderate to strong destabilizing effects on binding tend to reside along the pentameric protein-protein interfaces (red sites in Figure 2 A–B) and not along the edges that would form the between-pentamer interfaces.

**Figure 3:**
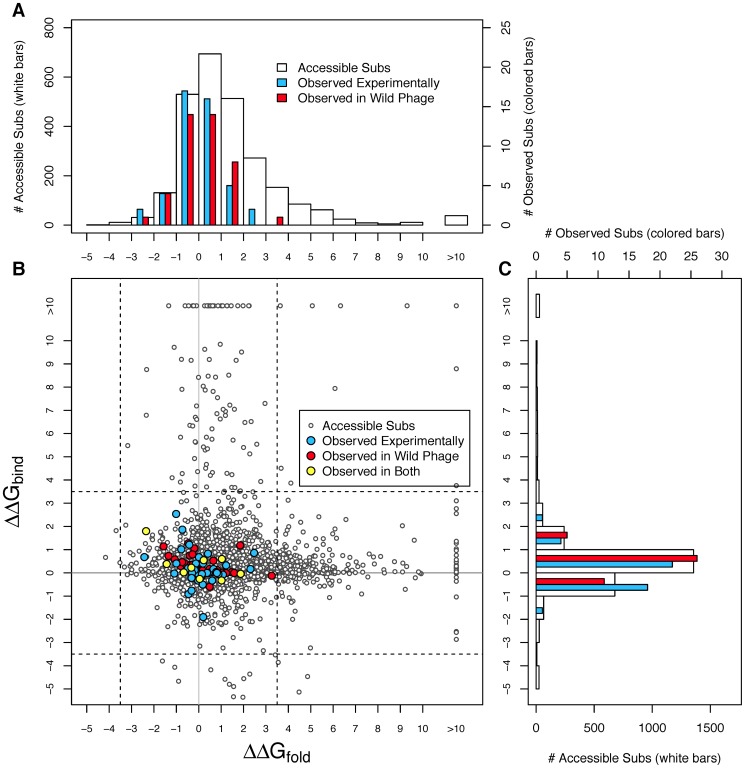
Comparison of stability effects between those accessible and those observed in the experimental and wild phage data. The figure shows that all observed substitutions have small effects on both folding and binding stability. (A) Histogram of *ΔΔG_fold_*. (B) Scatterplot of *ΔΔG_fold_* vs *ΔΔG_bind_*. (C) Histogram of *ΔΔG_bind_*. The dotted lines in (B) highlights the zone within which all observed substitutions fall. Note differences in scale between substitutions accessible (white bars) and those observed (red and blue bars) in the two histogram panels.

Examining the distribution of *ΔΔG_fold_* and *ΔΔG_bind_* of accessible substitutions shows that while most substitutions are destabilizing, they also tend to have small effects on stability (the white histogram bars in Figure 3A and C show accessible substitutions). For folding stability, 72.6% of the substitutions are between −2 and +2; for binding 91.1% are in this zone. If we had we included between pentamer-pentamer interactions, we expect that some of the substitutions along these interfaces would have been destabilizing and the distribution of *ΔΔG_bind_* would be more spread out, like that of *ΔΔG_fold_*. Finally, the scatterplot of in Figure 3B shows that there is no correlation between *ΔΔG_fold_* and *ΔΔG_bind_* (r^2^ = 0.0003, p = 0.39). This is not surprising given that substitutions having moderate to strong effects on binding stability occur at different residues than those having significant effects on binding stability (Figure 2).

### Patterns among Observed Substitutions

We next characterized changes in stability for substitutions that have been observed in real evolving populations: either substitutions implicated by a comparison of the φX174-like wild phage, or substitutions observed during laboratory adaptations of φX174. We find that while observed substitutions can be stabilizing or destabilizing, none of them have large effects on stability (in Figure 3, colored histogram bars and points are observed substitutions). When the two datasets are combined, 79 unique substitutions are observed. Of these, 74 (93.7%) have *ΔΔG_fold_* between −2 and +2, and 78 (98.7%) have *ΔΔG_bind_* between −2 and +2 (Figure 3; Table 1). The six substitutions that fall outside this zone are not far outside it, with the largest deviation being +3.26 kcal/mol. The values for the two datasets viewed separately are quite similar but with smaller sample sizes (Table 1).

**Table 1:** The proportion of substitutions with *ΔΔG* within a stability zone around zero and the associated p-values.

			*ΔΔG_fold_*		*ΔΔG_bind_*		*ΔΔG_fold_ & ΔΔG_bind_*	
Stability Zone	Set of Substitutions	n	Prop (n)	p-value	Prop (n)	p-value	Prop (n)	p-value
−2 to +2	Accessible	2570	0.726 (1866)	–	0.911 (2340)	–	0.660 (1696)	–
	Experimental	46	0.913 (42)	0.0038	0.978 (45)	0.1456	0.891 (41)	0.0003
	Wild phage	42	0.952 (40)	0.0002	1.0 (42)	0.0364	0.952 (40)	<0.0001
	Experimental + Wild	79	0.937 (74)	0.0002	0.987 (78)	0.0114	0.924 (73)	<0.0001
−3.5 to +3.5	Accessible	2570	0.879 (2260)	–	0.950 (2441)	–	0.835 (2146)	–
	Experimental	46	1 (46)	0.0048	1 (46)	0.1876	1 (46)	0.0001
	Wild phage	42	1 (42)	0.0068	1 (42)	0.2292	1 (42)	0.0006
	Experimental + Wild	79	1 (79)	<0.0001	1 (79)	0.0310	1 (79)	<0.0001

Stability zone defined in the left column. The top row within each stability zone shows the accessible substitutions against which the other sets are compared. For *ΔΔG_fold_*, *ΔΔG_bind_*, and *ΔΔG_fold_* & *ΔΔG_bind_* together, the left column gives the proportion of substitutions in the stability zone with the actual number in parentheses. The right column gives the p-value associated with the null hypothesis that the observed counts (experimental, wild, and experimental + wild rows) are random samples from the accessible set and fall in the stability zone by chance. By *ΔΔG_fold_* & *ΔΔG_bind_* we mean the substitutions observed jointly within the zone by both measures of stability. Test are all two-sided and based on 10,000 random samples of accessible set.

We conducted a randomization test to assess whether the observed substitutions differ significantly from the accessible substitutions. The answer is yes, observed substitutions are more concentrated near *ΔΔG_fold_* = 0 and *ΔΔG_bind_* = 0 than expected by chance. To perform the test, we took sets of 10,000 random samples from the accessible substitutions at the sample size of each observed set and asked how often the random sample has as many or more substitutions in the −2 to +2 stability zone as were actually observed. The test was done for folding stability alone, binding stability alone, or both folding and binding jointly. For the experimental and wild phage combined dataset, the two-sided p-values for folding, binding and the two jointly are 0.0002, 0.0114, and <0.0001 respectively (upper half of Table 1). For the two datasets individually, the smaller sample sizes lead to larger p-values, but except for binding in the experimental set, they remain significant. To check for robustness, we reran this test with the stability zone expanded to −3.5 to +3.5 and the results are very similar (Table 1).

The finding that observed substitutions differ from those accessible implies that selection acts on stability, either because stability or a trait highly correlated with it effects fitness or because the substitutions available to selection are constrained by their stability effects. We were interested in what selection surface could account for the differences between accessible and observed substitutions. To answer this, we assumed a simple model where that the probability of observing a substitution with a particular *ΔΔG_fold_*, *ΔΔG_bind_* value in the data is proportional to the density of accessible substitutions in this stability region multiplied by the density of a selection function at this point. We assumed the selection function was a bivariate normal truncated below −3 and above +3 in both stability dimensions. We then determined what parameter values would make the observed data most probable. Before examining the results, it is helpful to consider interpretation of several of the most extreme possible selection functions. A very flat, plateau-like, selection function corresponds to stability acting purely as a filter, indifferent to the stability effects except whether they fall within the truncated zone or not. By contrast, a tight and perfectly symmetrical peak at zero would indicate selection strongly favors substitutions that change neither folding nor bindings stability. A long narrow ridge running along one axis indicates selection is indifferent to the stability the ridge is along but very sensitive to the other type of stability.

**Figure 4:**
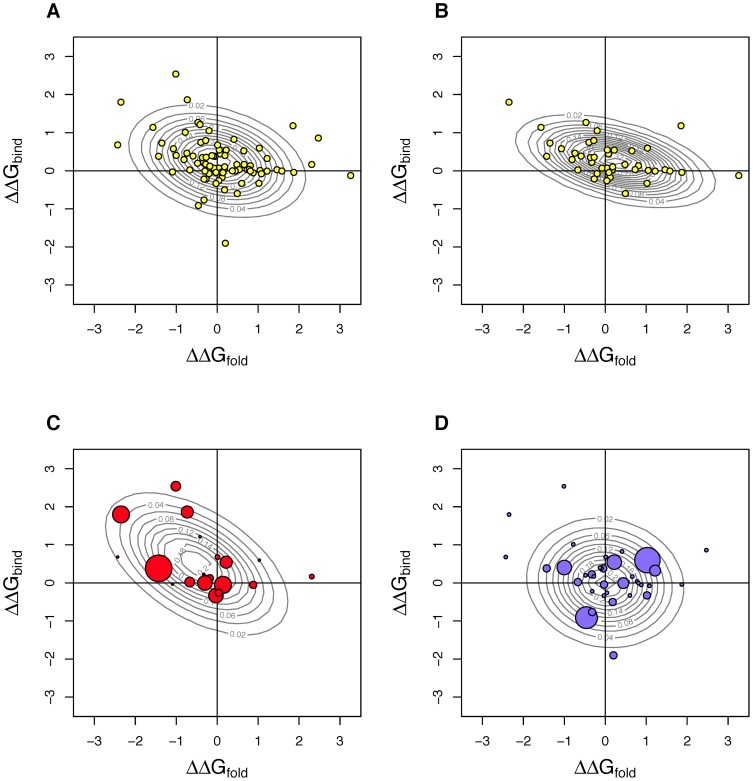
Estimated selection functions explaining the difference between accessible and observed substitutions. The figure shows that selection generally favors substitutions that have little effect on stability (peaks near the origin), but at high temperatures (in red), selection also favors substitutions that simultaneously stabilize folding and destabilize binding. The selection function is assumed to be a bivariate normal, the contour lines of equal probability of which are shown. Observed substitutions are colored circles. A) All 79 substitutions from both datasets combined. B) Wild phage dataset. C) Experimental data at high temperatures. D) Experimental data at normal temperature weighted by number of experiments observed in. In C and D substitutions are weighted by the number of experiments they appear in; size of symbols are scaled to show weighting. Density of accessible substitutions is shown in Figure 3B.

The best-fit selection functions are shown in Figure 4 with separate panels for the entire dataset combined, for the wild phage dataset, and the experimental datasets at high and normal temperatures. Averaging over the many conditions represented by our entire dataset (panel A), the selection function is centered on the origin indicating that selection favors substitutions that alter stability very little. The wild phage (panel B) are similar. The most interesting comparison is between the selection surfaces at high vs. normal temperatures (panels C and D). At high temperatures, the surface is a slightly elongated ridge running from the upper left quadrant down to the origin. In other words, selection favors substitutions with either little effect on stability or on those stabilize folding of the F protein and simultaneously destabilize binding of the pentamer (negative *ΔΔG_fold_* and positive *ΔΔG_bind_*). At normal temperature, we see a selection surface that is roughly circular with a peak very near the origin.

A possible interpretation of these results is that the F protein is either at or is close to its optimal stability. This view asserts that at normal laboratory temperature substitutions conferring small changes to stability may be neutral or beneficial, but those that result in large changes are deleterious. The same is true at high temperatures except that the optimum stability appears to be slightly shifted from the ancestor. At both temperatures, all the changes we observe in stability across temperatures are small (<2.5 kcal/mol). If this assertion that the protein is near or at the stability optimum is correct, we expect that the cumulative *ΔΔG_fold_* and *ΔΔG_bind_* over the course experiments (i.e. the sum *ΔΔG_fold_* and *ΔΔG_bind_* for all substitutions found in an experiment) should also remain in the same zone as individual substitutions. By contrast, if cumulative *ΔΔG_fold_* and *ΔΔG_bind_* depart from this region, then we know selection is limiting the size of individual stability changes while still allowing larger shifts the protein's stability.

We tested these competing possibilities by looking at cumulative *ΔΔG_bind_* and *ΔΔG_fold_* in laboratory adaptation as a function of temperature. Temperature is a good candidate for examining this question for several reasons. First, it has a profound effect on fitness, so selection is strong. Second, certain substitutions are observed repeatedly at high temperatures (e.g. L242F in Bull et al. 2000 [21]) indicating that they are adaptations to high temperature per se. Third, it is logical that protein stability links temperature to fitness since temperature affects stability, stability dictates the proportion of time the protein is folded and bound (as compared to unfolded and unbound), and we expect these proportions to affect viral assembly rate and therefore fitness.

**Figure 5:**
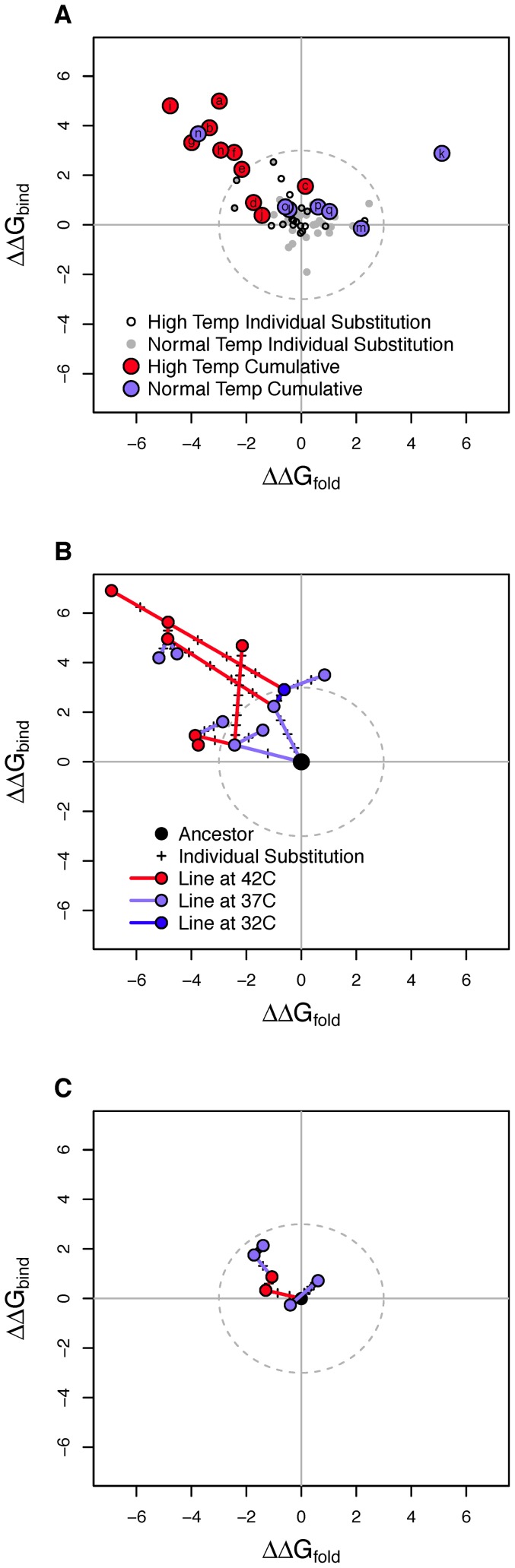
Cumulative changes in *ΔΔG_fold_* and *ΔΔG_bind_* across many lab adaptation experiments. The figure shows that cumulative stability changes frequently extend beyond individual changes and that high temperature changes are frequently beyond the range of individual changes and high temperatures (red) often push stability toward negative *ΔΔG_fold_* and positive *ΔΔG_bind_*. A) Colored symbols show cumulative stability changes for all experiments beginning with ancestral φX174 and remaining at either high (≥42°C) or low (≤37°C) temperatures. Small open and grey points show the stability changes for individual substitutions. The dashed circle demarks the range within which all single substitutions fall. Letters within colored symbols indicate from where the experiment data is obtained (see end of legend). B) Cumulative stability changes in the Rain experiment [41]. The experiment had a branching design where temperature differed between each of the two branches as indicated. Number of substitutions on each branch indicated by + symbols. C) Cumulative stability changes in two unpublished 50-day chemostat experiments that were sampled every 10 days where temperature began at 37°C, was elevated to 42°C for part of period of time, and then returned to 37°C. The letters in panel A indicate the study where each dataset comes from: a–b [43], c–d [49], e–I [8], j [45], k [46], l [49], n-o [48], and q [47]. Experiments m and p are unpublished.

The results, presented in Figure 5, show that the cumulative effects on stability often take the protein well outside the region where individual changes are found. If we look at adaptations that began with our ancestor (panel A), 7 of the 10 high temperature adaptations have cumulative effects outside the region of individual effects (denoted by the dashed circle). The most extreme case has *ΔΔG_fold_*≈−5 and *ΔΔG_bind_*≈5, roughly twice the magnitude of departure from ancestor observed among the largest individual changes. At normal laboratory temperature, two of the seven experiments depart from the region of individual effects, but each in a different manner. In panel B we present the results from an experiment where adapting lines were split repeatedly, with each branch subjected to different hosts and/or temperatures [41]. Similar to panel A, we observe high temperatures tending to shift stability up and to the left. Here, the most extreme endpoint falls at *ΔΔG_fold_*≈−7 and *ΔΔG_bind_*≈7, nearly three times the deviation found among individual changes. In panel C we show the results from two unpublished 50-day chemostat adaptations where temperature was initially normal (37°C), then high (42°C), and then returned to normal; populations were sampled every 10 days. For both populations we see only small cumulative changes, well within the range of individual effects.

Taken together, the cumulative *ΔΔG* results point to a few general conclusions. First, there is a lot of variation in the trajectory that stability takes under either temperature. This implies that selection must be acting on phenotypes beyond protein stability in these experiments. Second, cumulative changes can be much larger than individual changes. Because larger stability-changing substitutions are accessible, this suggests that selection favors several local modifications to stability over one large change that accomplishes the same thing at a global scale. Third, at high temperatures the stability trajectory tends to be toward negative *ΔΔG_fold_* and positive *ΔΔG_bind_*. The negative change in *ΔΔG_fold_* may be a way of counterbalancing the destabilizing effects of elevated temperature and leaving the protein highly functional.

The trend toward positive changes in *ΔΔG_bind_* are, however, quite unexpected. In previous work on the related bacteriophage ID11 [22], [23], we found the opposite patterns: a set of first-step substitutions that were highly beneficial at 37°C tended to stabilize binding (i.e. have negative *ΔΔG_bind_* values). There are several differences between the ID11 study and the φX174 experiments reported here. Most importantly, while 37°C is near the optima for φX174, the optima for ID11 is around 32°C [24], [25]; thus 37°C is a high temperature for ID11. Secondly, all of the changes reported for ID11 were first-step changes while each φX174 experiment reported accumulated many changes. Finally, those ID11 substitutions arose in flask adaptations where accessible hosts greatly outnumbered phage. Nearly all of the φX174 adaptations occurred in chemostats where hosts greatly outnumbered by phage.

In flasks, logic dictates that a good strategy is to minimize the time to burst (and thereby allow subsequent infections and rapid exponential growth) while in chemostats it should pay to maximize the number of progeny in the current infection. Indeed, chemostat adaptations of φX174 commonly have mutations is in the D-promoter that serve to delay the time to burst [2], [19], [26], [27]. One possible explanation for the tendency to destabilize binding at high temperatures is that this may slow capsid assembly. At high temperatures, cell growth is slowed and other aspects of phage reproduction like genome replication, translation and scaffolding construction are probably slowed as well. Slowing pentamer binding might bring the rate of capsid assembly into closer balance with other processes and ultimately increase burst size.

### Summary

We have shown that in major capsid protein of φX174, the majority of accessible substitutions destabilize both protein folding and pentamer binding. The substitutions that are observed in the wild phage and in laboratory adaptations of φX174 have significantly smaller effects on stability than expected. However, in adaptations to temperatures above 42°C, there is tendency for substitutions to accumulate that confer stabilizing effects on folding, but destabilizing effects on binding. One possibility is that these changes leave F still functional, but slow the rate of pentamer and thereby capsid assembly in a way that increases burst size. Finally, the cumulative stability effects over the course of an adaptation are often greater than the range of individual changes suggesting that there are local as well as global constraints on protein stability.

## Materials and Methods

### Phage System

The organism used for this study is phage φX174, a virus that infects *Escherichia coli* and other bacteria [23]. Phage φX174 has 11 genes and is composed of several proteins depending on the stage of the assembly cycle [33]. The φX174 mature capsid (Figure 1A) is composed of 12 pentameric units containing proteins F, G, and J, plus 12 copies of H asymmetrically arranged inside the capsid [34]–[37]. The model system for the current study is the coat protein F which must both fold and then bind to form pentameric subunits in the early stage of the procapsid formation (Figure 1B; Figure 1C).

### Stability Estimation

Changes in protein folding stabilities and protein-protein binding stabilities due to amino acid substitutions were estimated using FoldX [7]. FoldX was chosen for this study to balance accuracy and speed [3], [5]–[7]. Given the large number of mutations studied here, it is not possible to use accurate statistical mechanical approaches such as all atom molecular dynamics simulation as we did in a previous study [23]. A total of 2570 substitutions (all substitutions at the 426 residues of protein F accessible with one DNA mutation) were estimated for each protein structure in unbound and pentameric system (Figure 1B, 1C). Initially, protein structures were equilibrated 15 times in succession using the “repairPDB” command in the FoldX software to obtain a fully minimalized conformation. Once the minimized conformation was obtained for each of the four model systems, then the binding and folding stabilities were estimated using the “BuildModel” command in FoldX (also see Figure 3). The estimated folding and binding stability changes for all possible single substitutions from the reference sequence are available in the supplemental materials.

### Observed Substitutions

Observed substitutions came from two different datasets: wild and experimental. The wild phage substitutions were based on the collecting, sequencing and phylogenetic work of Rokyta et al. [12] We obtained the F-protein amino acid sequences for 19 phage in the φX174-like clade, including φX174 itself. We used the consensus sequence of these to generate a putative ancestral sequence. Comparison of the 19 phage with this ancestral sequence yielded 42 unique substitutions among the wild phage. For the experimental set we constructed a database of many published [8], [41], [43]–[50] and two unpublished laboratory adaptations involving φX174. The dataset includes a total of approximately 29 different experiments (the count is complicated by the fact that some experiments involved branching lines). All but five of the experiments were conducted in chemostats (the others were in flasks); 17 of them began with our ancestor φX174 (the others used φX174 with substitutions already in the genome); 12 of them were at high temperatures (42–43.5°C), 13 at normal laboratory temperature (37°C), while 4 of them involved variable temperatures. Normal laboratory temperature is close to the optimal for φX174, while these high temperatures constitutes strong selection on this phage [15].

### Statistical Analysis

To determine whether the observed substitutions were more narrowly clustered around *ΔΔG* of zero than expected, we did a set of randomization tests. We fist defined a zone around zeros as −2 to +2. We defined n_fold(real)_, n_bind(real)_ and n_fold+bind(real)_ as, respectively, the number of real observed substitutions with *ΔΔG_fold_* individually *ΔΔG_bind_* individually, and *ΔΔG_fold_* and *ΔΔG_bind_* simultaneously inside this zone. For the wild phage, we drew samples of size 42 (the number of observed substitutions) without replacement from the pool of accessible substitutions and, each time, determined the number of substitutions within the zone by each criteria: n_fold(sim)_, n_bind(sim)_ and n_fold+bind(sim)_. We did this 10,000 times and approximated p-values as twice the proportion of times the n_fold(sim)_≥n_fold(real)_, n_bind(sim)_≥n_bind(real)_, and n_fold+bind(sim)_≥n_fold+bind(real)_. We then repeated this for the set of 46 experimentally observed substitutions, and the combined set of 79 substitutions. Finally, we redefined the zone as −3.5 to +3.5 and reran the analyses.

We estimated selection functions that could explain the disparities between accessible and observed substitutions. To do this we assumed that the approximate probability of observing a substitution in the data with a particular joint *ΔΔG_fold_* and *ΔΔG_bind_* value was proportional to the product of the density of accessible substitutions in this stability region and the density of the selection function at this point. The accessible densities were obtained by gridding the region between −3 and +3 at 0.25 increments and calculating the proportion of accessible substitutions within each square. We considered candidate bivariate normal distributions across a range of parameter values: μ_fold_ and μ_fold_ from −1 to +1 at 0.1 increments, σ_fold_ and σ_bind_ from 0.25 to 1.5 at 0.0625 increments, and ρ from −1 to +1 at 0.1 increments. For each we obtained the density at that *ΔΔG_fold_*, *ΔΔG_bind_* value, multiplied by accessible density in that region, took the log, and summed over all substitutions in the dataset. The combination of parameter values that made this sum largest served as our estimated of the selection function. We did this for wild dataset alone, for the combined wild plus experimental dataset, for the experimental data at 37°C, and the experimental data at 42–43.5°C. In the last two cases we restricted ourselves to experiments that began with ancestral φX174 (excluding those that had previous adaptive changes). For these, we have ran the analysis both with each substitution represented once (unweighted) and with each substitution weighted by the number of different experiments it appeared in. We present the results from the weighted analysis, but the unweighted results were qualitatively the same.

### Accession Numbers

The ancestral φX174 sequence is available at GenBank accession number AF176034. The model structure is based on Protein Data Bank accession number 2BPA.

## Supporting Information

Table S1 **FoldX estimates of** ***ΔΔG_fold_*** **and** ***ΔΔG_bind_*** **for all 8094 possible single substitutions in the φX174 F protein relative to the reference sequence.** *site* is the residue number. Note in protein F the first amino acid, methionine, is removed after translation. Numbering begins after its removal. *aa.from* and *aa.to* are the amino acids in the reference and the mutant respectively. *within.1.DNA.change* indicates substitutions that can be accessed by a single DNA change from the reference sequence (1 =  yes, 0 =  no). *wild.phg.sub* indicates substitutions we infer occurred in the evolution of the φX174-like wild phage by comparison of them with their consensus sequence (1 =  yes, 0 =  no). *lab.exp.sub* indicates substitutions found in a lab adaptation experiment (see paper for source of experiments; 1 =  yes, 0 =  no). *ddG.fold* and *ddG.bind* give changes in folding and binding stability, *ΔΔG_fold_* and *ΔΔG_bind_*, respectively.
(TXT)
